# *Ascaris lumbricoides* Infection and Its Relation to Environmental Factors in the Mbeya Region of Tanzania, a Cross-Sectional, Population-Based Study

**DOI:** 10.1371/journal.pone.0092032

**Published:** 2014-03-18

**Authors:** Steffen Andreas Schüle, Petra Clowes, Inge Kroidl, Dickens O. Kowuor, Anthony Nsojo, Chacha Mangu, Helene Riess, Christof Geldmacher, Rüdiger Paul Laubender, Seif Mhina, Leonard Maboko, Thomas Löscher, Michael Hoelscher, Elmar Saathoff

**Affiliations:** 1 Division of Infectious Diseases and Tropical Medicine, Medical Center of the University of Munich (LMU), Munich, Germany; 2 Institute for Medical Bioinformatics, Biometry, and Epidemiology, Ludwig-Maximilians-University, Munich, Germany; 3 Department of Social Epidemiology, Institute for Public Health and Nursing Research, University of Bremen, Bremen, Germany; 4 NIMR-Mbeya Medical Research Center (MMRC), Mbeya, Tanzania; 5 German Center for Infection Research (DZIF), partner site Munich, Munich, Germany; 6 Mbeya Regional Medical Office, Mbeya, Tanzania; Tulane University School of Public Health and Tropical Medicine, United States of America

## Abstract

**Background:**

With one quarter of the world population infected, the intestinal nematode *Ascaris lumbricoides* is one of the most common infectious agents, especially in the tropics and sub-tropics. Infection is caused by oral intake of eggs and can cause respiratory and gastrointestinal problems. To identify high risk areas for intervention, it is necessary to understand the effects of climatic, environmental and socio-demographic conditions on *A. lumbricoides* infection.

**Methodology:**

Cross-sectional survey data of 6,366 study participants in the Mbeya region of South-Western Tanzania were used to analyze associations between remotely sensed environmental data and *A. lumbricoides* infection. Non-linear associations were accounted for by using fractional polynomial regression, and socio-demographic and sanitary data were included as potential confounders.

**Principal Findings:**

The overall prevalence of *A. lumbricoides* infection was 6.8%. Our final multivariable model revealed a significant non-linear association between rainfall and *A. lumbricoides* infection with peak prevalences at 1740 mm of mean annual rainfall. Mean annual land surface temperature during the day was linearly modeled and negatively associated with *A. lumbricoides* infection (odds ratio (OR) = 0.87, 95% confidence interval (CI) = 0.78–0.97). Furthermore, age, which also showed a significant non-linear association (infection maximum at 7.7 years), socio-economic status (OR = 0.82, CI = 0.68–0.97), and latrine coverage around the house (OR = 0.80, CI = 0.67–0.96) remained in the final model.

**Conclusions:**

*A. lumbricoides* infection was associated with environmental, socio-demographic and sanitary factors both in uni- and multivariable analysis. Non-linear analysis with fractional polynomials can improve model fit, resulting in a better understanding of the relationship between environmental conditions and helminth infection, and more precise predictions of high prevalence areas. However, socio-demographic determinants and sanitary conditions should also be considered, especially when planning public health interventions on a smaller scale, such as the community level.

## Introduction

The intestinal nematode *Ascaris lumbricoides* is one of the most common causes of infection among the soil-transmitted helminths (STH). Common in the tropics and sub-tropics, it is estimated that more than one quarter of the world population is infected with this helminth [1]–[3].

The highest morbidity is found in children, especially in those with a high worm burden. *A. lumbricoides* can lead to reduced physical fitness, growth retardation, and respiratory and gastrointestinal problems [3]–[6]. Evidence if *A. lumbricoides* infection has a negative impact on cognitive function and educational achievement in school children is controversially debated [7]–[10].

Infection occurs through the oral intake of eggs, usually contained in soil or food. Adult worms live in the lumen of the small intestine where the female lays unembryonated eggs which are excreted with the feces. In the open, the eggs have to go through three stages of development in order to become infectious; a time during which they are exposed to environmental conditions [5], [11], [12]. When embryonated eggs are swallowed by a human host, the larvae hatch in the small intestine, have a short migratory phase (venous system, liver, lungs, trachea, esophagus) after which they return to the small intestine where they mature and mate [13], [14].

Recently, remotely sensed environmental data have increasingly been used to get a better understanding of the epidemiology and spatial distribution of STH [15]–[22]. The use of environmental data in combination with geographic information systems (GIS) has become a powerful tool for mapping and predicting STH, with the main purpose to identify high risk areas for intervention [23]–[26].

However, there are still challenges in the statistical modeling of environmental data. One problem is the consideration of non-linear relationships between outcome and predictor variables. Although non-linear relationships between environmental data and STH infection are a recognized fact [16], [22], this has rarely been taken into account in multivariable analysis. A further complication is the need to take care of potential confounders. Especially risk factors linked to transmission, such as poor sanitation facilities, crowding, and high population density [2], [6], [12], [14], [27]–[32], need to be considered when associations between environmental factors and STH infection are analyzed.

Therefore, the main objective of this study was to assess associations between remotely sensed environmental data and *A. lumbricoides* infection while considering potential non-linear relationships and confounders. A manuscript that examines associations of hookworm infection with environmental factors has recently been accepted [33], and manuscripts regarding *Trichuris trichiura* and schistosome infection are presently being prepared.

## Methods

### Ethics Statement

The study was approved by the ethics committee of the Tanzanian National Institute for Medical Research and conducted according to the principles expressed in the Declaration of Helsinki. All participants provided written informed consent before enrolment into the study; parents consented for their children below 18 years of age. Specifically, children who were old enough to understand the process were asked to participate in the consenting procedure, and children who were 12 years old or older were asked to sign/thumbprint the document in addition to their parent's signature/thumbprint.

### Study Area and Epidemiological Data Collection

**Figure 1:**
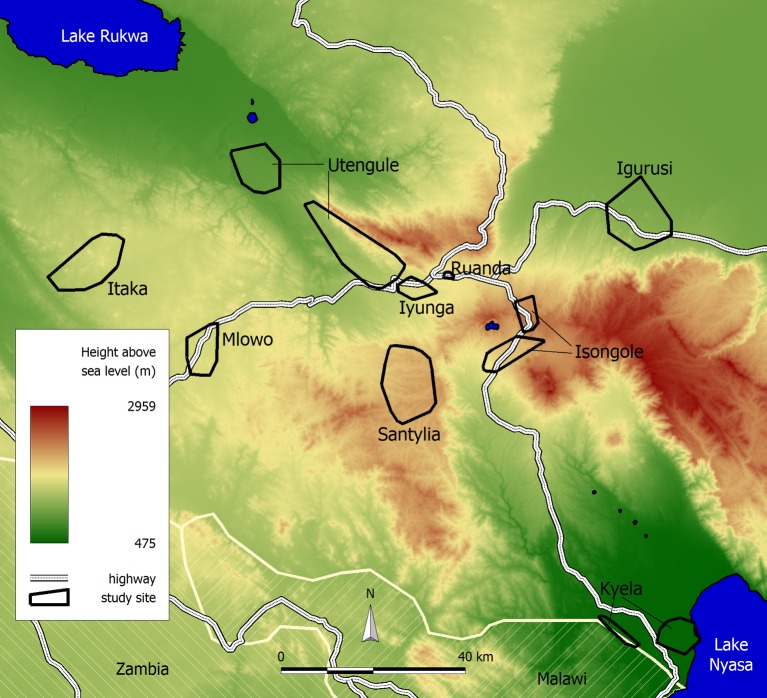
Location and altitude of the EMINI study sites. The large altitude range results in very diverse environmental conditions regarding temperature, vegetation, slope etc.

Data for this study were collected in nine study sites in the Mbeya Region in south-western Tanzania (Figure 1) from June 2008 until June 2009 during the third annual survey of the EMINI (Evaluating and monitoring the impact of new interventions) cohort study. The region is predominantly rural and most income generating activities are related to agriculture. During an initial population census in the nine sites, more than 42,000 households were identified and their geographical positions recorded, using handheld geographical positioning system (GPS) devices (SporTrak handheld GPS, Magellan Navigation Inc., Santa Clara, CA, USA). Geographically stratified random selection was used to choose 10% (4,283) of these households to participate in the main EMINI cohort study. During each annual survey these households were visited once to collect biological specimen and interview data. All participants provided written informed consent before inclusion into the study, with parents or care takers consenting for their minors.

The collection of stool samples started in 2008. Due to logistic constraints, households were randomized into two groups of equal size of which only one was annually sampled for stool.

Interviews to characterize the socio-economic status (SES) of each household were conducted with the household head and included questions regarding infrastructure of the household, ownership of livestock, the availability of certain household assets, and materials that were used to build the house(s) in each homestead. Data on socio-demographic status (sex, age, marital status, religious denomination, education, occupation etc.), relevant behavior, knowledge and practices regarding various diseases were collected in interviews with each individual household member or – for children below 12 years – with their caretaker. All interviews and medical examinations were performed at the household and conducted in Kiswahili language.

Before stool collection started in the third survey round, intestinal nematodes were neither diagnosed nor treated as part of this study, and to our knowledge no other treatment programs had been conducted in the region. Stool samples were collected in pre-labeled screw-top containers, refrigerated at 4 °C directly after collection, using mobile refrigerators (WAECO CoolFreeze CF-50, WAECO, Emsdetten, Germany) and kept cool until slide preparation in the laboratory within two days of collection. The *A. lumbricoides* infection status of participants was established by Kato-Katz examination [34] of two sub-samples (41.7 mg each) from a single stool specimen, which was thoroughly mixed before slide preparation. Kato-Katz slides were examined for *A. lumbricoides* eggs by experienced staff within two days after slide preparation. *A. lumbricoides* infection was defined as the presence of at least one *A. lumbricoides* egg in any of the two slides and infection intensity was classified according to Montresor et al. [35]. To assure the quality of our lab results all Kato-Katz slides were archived and a sample of randomly selected slides were reexamined after at least one month by different lab staff.

Helminth infected participants were offered treatment with albendazole (for *A. lumbricoides* and other intestinal nematode infections) and/or praziquantel (for schistosome infections), according to their respective diagnosis.

### Environmental Data

The following remotely sensed environmental data were considered for this analysis: Elevation was obtained using the NASA Shuttle Radar Topography Mission (SRTM) global digital elevation model (DEM) version 2.1 [36]. These elevation data were also used to calculate the slope. Mean annual rainfall and ambient temperature were downloaded from the WorldClim – Global Climate Data website [37]. Mean annual land surface temperature during day and night (LST-day and LST-night) and vegetation cover (Enhanced vegetation index (EVI)) which had been collected during NASA's Moderate-Resolution Imaging Spectroradiometer (MODIS) Terra mission, were downloaded from the Land Processes Distributed Active Archive Center (LP DAAC) [38], [39].

Household positions and inhabitant numbers from the initial population census were used to calculate population density around the household. Population density, ambient temperature, elevation, rainfall, LST, EVI, and slope were averaged for a buffer area within a 1,000 meter radius around each homestead in order to characterize the environmental situation around the household. This approach was preferred to using the respective spot values at the homestead position because spot data are more prone to random error than averages for a wider area. Latrine coverage in the surroundings of each household was calculated as the inverse distance weighted percentage of households with their own latrine within one kilometer around the household.

Initial processing of remotely sensed data was done in Idrisi GIS software v.32 (Clark Labs, Worcester, MA, USA). The GIS program Manifold System 8.0 Professional Edition (Manifold Net Ltd, Carson City, NV) was used to combine household positions and environmental data.

### Socio-economic Status and Other Confounding Variables

Household income and expenditure data in developing countries, especially in rural areas, are often unreliable because many people do not have a regular cash income. To overcome this problem we employed a modification of a method initially proposed by Filmer and Pritchett (2001) that uses principal component analysis to generate an SES score using proxy variables [40]–[42]. The following proxy variables were used: Household assets (clock or watch, radio, television, mobile telephone, refrigerator, hand cart, bicycle, motor cycle, car, savings account), construction materials for the house, and sources of household fuels and drinking water. In addition to the above described SES score, age, sex, population density, latrine coverage around the household, and presence of a latrine in the household were considered as potential confounders.

### Statistical Analysis

All statistical analyses were performed using Stata/SE (Version 11.2, StataCorp LP, College Station, TX). Because our environmental variables showed a high degree of correlation, multicollinearity was assessed using the variance inflation factor (VIF) (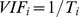) calculated with the tolerance (T) (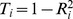). 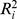 is the calculated variance of each covariate associated with the rest of the other independent variables. A VIF higher than 10 indicates a serious problem of multicollinearity [43]–[45].

After multicollinearity analysis we performed univariable linear logistic regressions for each considered independent variable. All variables with a univariable Wald's p<0.2 were included in the multivariable analysis.

In our study design individual observations were clustered in households and these were clustered within study sites. Therefore, household clustering was accounted for by calculation of robust standard errors using Huber/White variance estimates [46]–[48] and the nine study sites were taken into account as dummy variables.

Multivariable logistic regression with the inclusion of fractional polynomials which is a flexible parametric approach for modeling continuous factors was applied to analyze non-linear associations between *A. lumbricoides* infection and environmental variables [49]. The power transformations 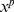 are found with a predefined set of powers S = −2,−1,−0.5,0,0.5,1,2,3 where 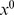 is defined as 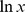. A fractional polynomial model with one degree (FP1) takes the form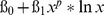, with two degrees (FP2)
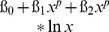
. The restriction of powers and the consideration of polynomials with degree 1 and 2 provide enough flexibility for statistical modeling [50], [51].

Multivariable fractional polynomial (MFP) models, an extended algorithm introduced by Royston and Sauerbrei, were used to detect non-linear associations. The MFP algorithm contains a function selection procedure which compares null, linear, and FP1 sub models for each covariate with an FP2 model based on the deviance [52]. A detailed description of the MFP algorithm is found in Ambler and Royston (2001) and in Sauerbrei and Royston (2008) [53], [54]. For the function selection procedure a lower p-value than 0.05 is recommended to avoid over fitting [50]. Therefore, a p-value of 0.01 was chosen as cut off when non-linear sub models were compared.

Our final model was calculated by removing variables with a p-value above 0.05. Changes of the Akaike Information Criterion (AIC) [55] and the Bayesian Information Criterion (BIC) [56], measuring the relative goodness of fit, were simultaneously considered. In order to asses spatial autocorrelation in the raw *A. lumbricoides* infection data and in the deviance residuals of our final logistic model the Stata module “spatcorr” was used to calculate Moran's I [57].

## Results

### Descriptive Results

**Figure 2:**
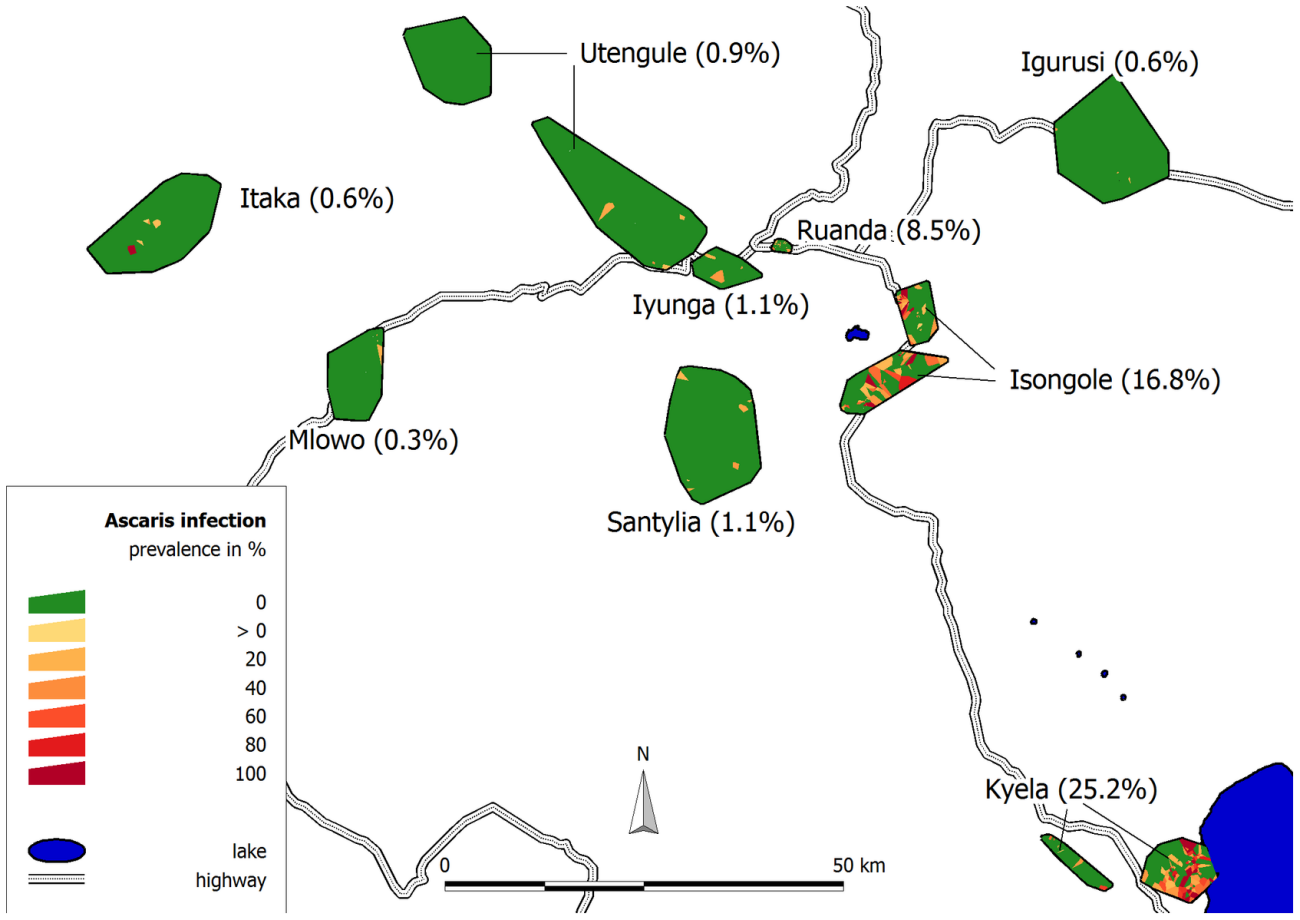
*A. lumbricoides* prevalence in the study sites. Color coding indicates household prevalence, labels indicate site name and site prevalence. *A. lumbricoides* infection is strongly clustered both between and within sites.

The overall prevalence of *A. lumbricoides* infection in the study population was 6.8% (n = 433/6,366). Most infections were of low intensity, moderate and high intensity infections were rare. The highest prevalences were found in Kyela (25.2%) and Isongole (16.9%). sites. Figure 2 demonstrates that *A. lumbricoides* infection was clustered both between and within sites.

**Figure 3:**
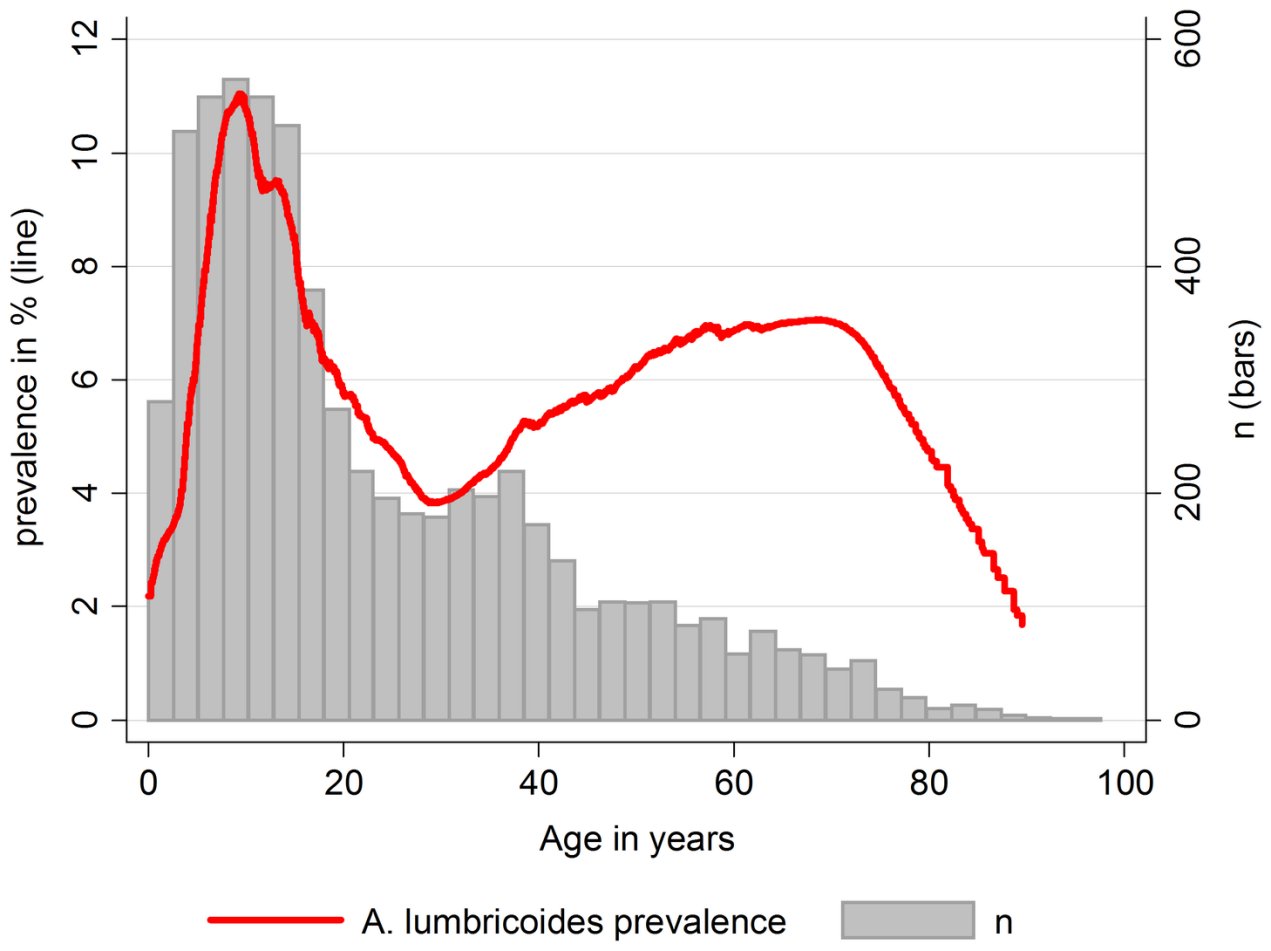
Lowess smoothed plot of unadjusted *A. lumbricoides* prevalence over age. The main prevalence peak in childhood is in accordance with the age of maximum infection intensity mentioned in the literature [14], [67]. The second rise above the age of 30 with a less pronounced peak in older age seems less common.

Men (47%) and women (53%) were almost equally represented in the study and the mean age was 23.6 years. Thus the majority of the study population were children and adolescents and the peak of *A. lumbricoides* infection occurred before the age of ten years (Figure 3). Nearly all households (97.5%) had their own latrine, which was a pit latrine in most cases (Table 1). The prevalence of *A. lumbricoides* infection was similar in female (6.57%) and male (7.11%) participants.

**Table 1:** Description of variables.

Variables	N	Mean or percentage a)	Std. Dev.	Min	Max
*Ascaris* infected	6,366	6.8%			
*Ascaris* infection intensity b):					
No infection (0 EPG)	5,933	93.2%			
Low intensity (1–1,999)	317	4.98%			
Moderate intensity (2,000–3,999 EPG)	66	1.04%			
Heavy intensity (≥4,000 EPG)	50	0.97%			
Elevation [m]	6,366	1455	486	479	2313
Mean annual ambient temperature [°C]	6,366	19.8	2.8	14.7	25.0
Mean annual rainfall [mm]	6,366	1437	378	1013	2342
Mean annual LST-day [°C]	6,366	32.4	2.5	22.5	38.6
Mean annual LST-night [°C]	6,366	14.5	3.4	9.2	21.4
Mean annual EVI	6,366	0.288	0.058	0.151	0.472
Slope [°]	6,366	3.03	2.21	0.35	13.64
Age [years]	6,366	23.6	19.2	0	97.7
Male gender	6,317	47.0%			
Household size [persons]	6,363	6.5	3.6	1	30
Population density [persons/km^2^]	6,366	1875	3179	10	13133
SES	6,363	−0.52	1.17	−2.82	4.08
Households with latrine	6,363	97.5%			
Latrine coverage in surroundings [%] c)	6,366	95.8	8.2	29.6	100

N =  number of observations; Std. Dev.  =  standard deviation; EPG  =  eggs per gram of feces; LST  =  land surface temperature; EVI  =  enhanced vegetation index; SES  =  socio-economic score.

a) Mean for continuous and % for categorical variables.

b) According to Montresor, 1998 [35].

c) Percentage of households with a latrine within one kilometer around the participant's household.

### Univariable Logistic Regression and Multicollinearity Analysis

In univariable analysis, all considered environmental variables were significantly associated with *A. lumbricoides* infection (Table 2). Elevation, LST-day and slope showed an inverse association, whereas all other environmental variables were positively associated. Therefore, all environmental variables apart from elevation and ambient temperature were included in the multivariable analysis. Sex, household size, and population density were excluded because their p-values were above 0.2, the threshold which was chosen for the inclusion in multivariable analysis.

**Table 2:** Univariable association of environmental and socio-demographic factors with *A. lumbricoides* infection a).

Variables	OR (95% CI)	p-value
Elevation, per 100 meters	0.88 (0.84 to 0.91)	<0.001
Mean annual ambient temperature, per 1°C	1.23 (1.14 to 1.32)	<0.001
Mean annual rainfall, per 1000 mm	7.93 (5.85 to 10.75)	<0.001
Mean annual LST-day, per 1°C	0.73 (0.70 to 0.76)	<0.001
Mean annual LST-night, per 1°C	1.20 (1.13 to 1.27)	<0.001
Mean annual EVI, per 0.1 units	5.91 (4.10 to 8.50)	<0.001
Slope, per 1 °	0.72 (0.63 to 0.83)	<0.001
Age, per 10 years	0.95 (0.90 to 1.00)	0.060
Sex	1.09 (0.90 to 1.32)	0.389
Household size	1.01 (0.96 to 1.07)	0.603
Population density, per 1000/km^2^	0.98 (0.94 to 1.03)	0.379
SES, per 1 unit	0.59 (0.48 to 0.73)	<0.001
Latrine in household (yes/no)	0.24 (0.13 to 0.46)	<0.001
Latrine coverage, per 10%b)	0.57 (0.50 to 0.65)	<0.001

OR  =  odds ratio; 95% CI  =  confidence interval; LST  =  land surface temperature; EVI  =  enhanced vegetation index; SES  =  socio-economic score.

a) Results of logistic regression models adjusted for household clustering using Huber/White variance estimates.

b) Percentage of households with a latrine within one kilometer around the participant's household.

Due to high multicollinearity of the variables elevation (VIF = 116.65), ambient temperature (VIF = 71.05) and LST-night (VIF = 17.19), elevation and ambient temperature were excluded from multivariable analysis, since their VIFs by far exceeded the threshold of 10. We decided to include LST-night not only because of its lower VIF, but more importantly, because soil temperature appears to be more directly linked to helminth egg development than elevation or ambient temperature, as eggs develop in the soil or at the soil surface.

### Multivariable Logistic Regression with Fractional Polynomials

In MFP analysis we found a non-linear relationship of rainfall and age with *A. lumbricoides* infection. In the full and in the final reduced model a fractional polynomial transformation with two degrees (FP2) was implemented.

For the other variables the linear assumption was retained and odds ratios (ORs) were calculated. The full and the final reduced multivariable regression models are shown in Table 3. Rainfall and LST-day were kept as significant environmental variables in the final model. LST-day showed an inverse association with *A. lumbricoides* infection. With every degree Celsius increase in LST-day the odds of being infected with *A. lumbricoides* decreased by about 13%.

**Table 3:** Multivariable association of environmental and socio-demographic factors with *A. lumbricoides* infection using logistic regression with fractional polynomials (n = 6,363).

	Full Model a)		Final Model after Backward Elimination a)	
Covariables	β/OR (95% CI)	p	β/OR (95% CI)	p
Mean annual rainfall, per 1000 mm	β_1_ = 1.92 (0.33 to 3.51)	0.018	β_1_ = 1.58 (0.14 to 3.02)	0.032
(FP2 polynomial transformationb))	β_2_ = −2.08 (−3.63 to −0.52)	0.009	β_2_ = −1.78 (−3.19 to −0.37)	0.013
Mean annual LST-day, per 1 °C	OR = 0.82 (0.73 to 0.93)	0.002	OR = 0.87 (0.78 to 0.97)	0.012
Mean annual LST-night, per 1 °C	OR = 1.24 (0.81 to 1.90)	0.315	.	.
Mean annual EVI, per 0.1 units	OR = 0.79 (0.42 to 1.50)	0.471	.	.
Slope, per 1 °	OR = 0.90 (0.72 to 1.12)	0.324	.	.
Age, per 10 years	β_1_ = 2.57 (1.38 to 3.76)	<0.001	β_1_ = 2.56 (1.38 to 3.74)	<0.001
(FP2 polynomial transformationc))	β_2_ = 1.13 (0.57 to 1.70)	<0.001	β_2_ = 1.13 (0.56 to 1.69)	<0.001
SES, per 1 unit	OR = 0.84 (0.70 to 1.00)	0.052	OR = 0.82 (0.68 to 0.97)	0.024
Latrine in household (yes/no)	OR = 0.68 (0.32 to 1.43)	0.310	.	.
Latrine coverage, per 10%d)	OR = 0.82 (0.68 to 0.99)	0.043	OR = 0.80 (0.67 to 0.96)	0.018
				
AIC	2178		2178	
BIC	2313		2287	

β =  beta coefficient; OR  =  odds ratio; 95% CI  =  confidence interval; LST  =  land surface temperature; EVI  =  enhanced vegetation index; SES  =  socio economic score, AIC  =  Akaike information criterion; BIC  =  Bayesian information criterion.

a) Adjusted for household clustering using Huber/White/Sandwich variance estimates and for study sites.

b) Fractional polynomial transformation with two degrees and powers p = 3: β_1_x^p^+β_2_x^p^*ln x.

c) Fractional polynomial transformation with two degrees and powers p = −0.5: β_1_x^p^+β_2_x^p^*ln x.

d) Percentage of households with a latrine within one kilometer around the participant's household.

**Figure 4:**
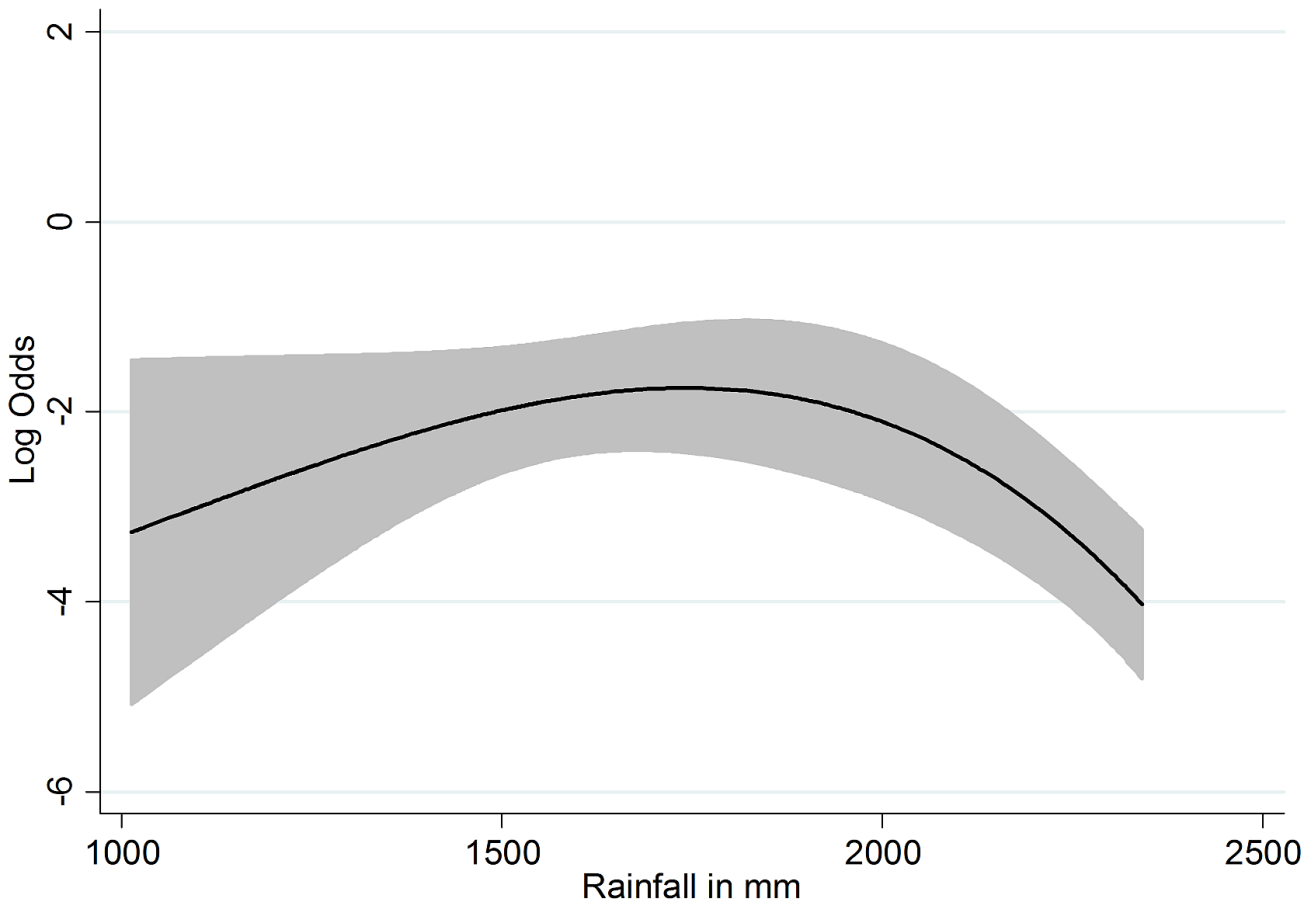
Non-linear partial prediction of the log odds of *A. lumbricoides* infection by annual rainfall. The partial predicted curve is adjusted for LST-day, slope, SES, age, latrine coverage and the nine study sites. The maximum is at 1740 mm of mean annual rainfall. Grey shadings indicate 95% confidence band.

The calculated beta coefficients for the non-linear functions of rainfall and age are not directly interpretable as ORs. For rainfall, the odds of *A. lumbricoides* infection are increasing until a rainfall maximum of 1,740 mm and are decreasing again at higher values (Figure 4). For age there is a steep increase until an infection maximum at 7.7 years, after which the odds are decreasing (curve not shown). This is in agreement with the unadjusted age prevalence curve shown in figure 2.

Except for latrine ownership in the household, all included confounding variables (age, SES score and latrine coverage) were significantly negatively associated with *A. lumbricoides* infection and therefore retained in the final multivariable model.

In order to check our final model for plausible interactions we calculated interaction terms between each beta of rainfall and LST-day and between SES and latrine coverage. However, none of these interactions were significant (data not shown).

**Figure 5:**
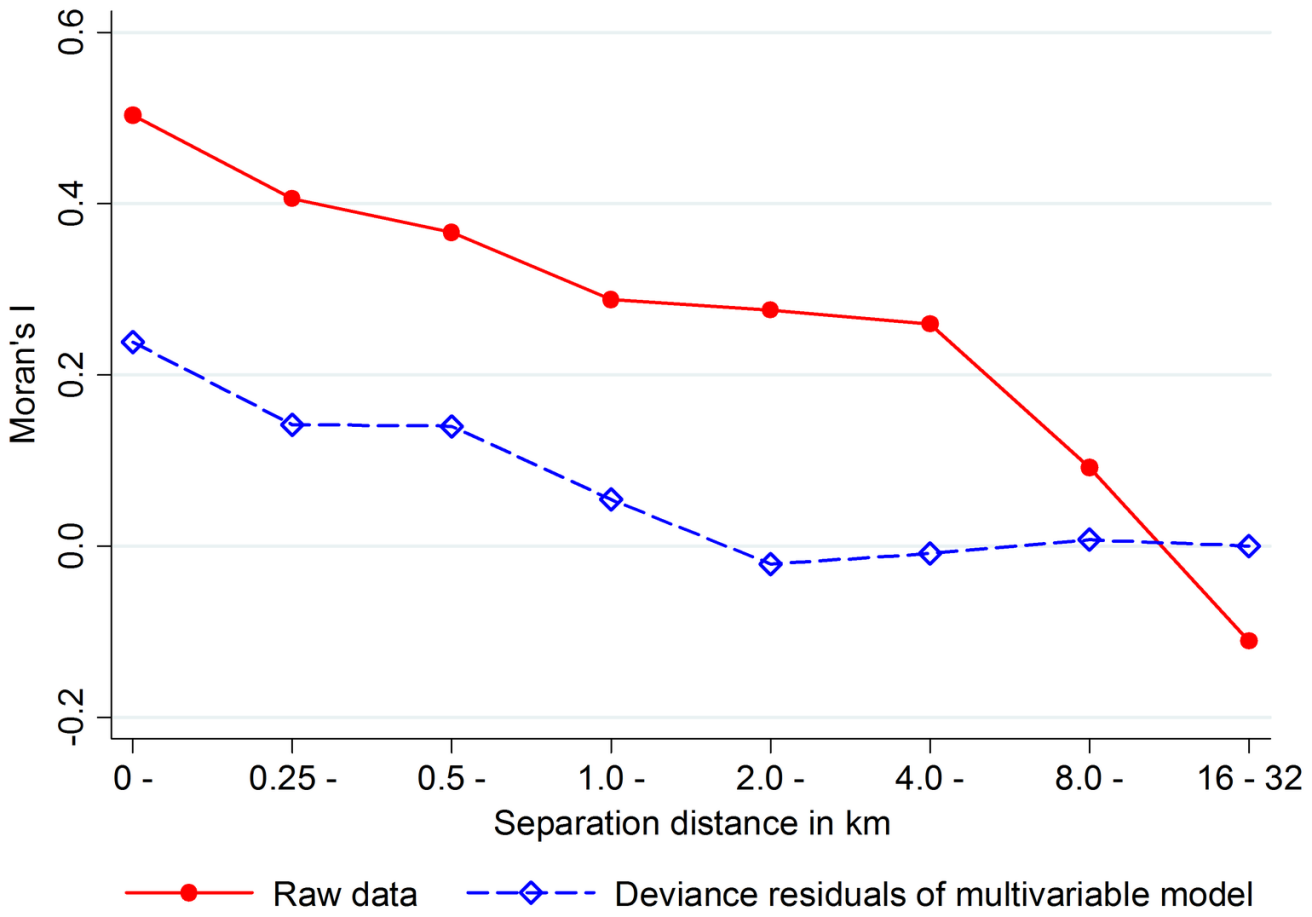
Spatial autocorrelation of *A. lumbricoides* infection within sites. Moran's I for spatial autocorrelation of *A. lumbricoides* infection in the raw data and in the deviance residuals of the final multivariable model. Values above 0 indicate positive, values below 0 negative spatial autocorrelation. The figure only considers autocorrelation between households within the same sites.

The raw *A. lumbricoides* infection data show strong positive spatial autocorrelation within separation distances of up to 8 km (Figure 5). The lower values for Moran's I in the deviance residuals of our final model indicate that the variables in the model account for a large part of this autocorrelation.

## Discussion

Our univariable results show that *A. lumbricoides* infection is significantly associated with several environmental factors. Of these, mean annual rainfall and mean annual LST-day remained significant in the multivariable model. LST-day had a linear negative association with *A. lumbricoides* infection, whereas the association of rainfall and age was non-linear with maximum infection odds at 1,740 mm of mean annual rainfall and at an age of 7.7 years. SES and latrine coverage around the household showed significant negative associations with *A. lumbricoides* infection, both in univariable and multivariable analyses.

Our results concerning LST-day are in line with the published literature [21], [23]–[25]. Two studies from Cameroon and Southeast Asia found that a higher LST was significantly associated with a lower risk of *A. lumbricoides* infection because high soil temperatures reduce humidity and thus lead to desiccation of *Ascaris* eggs [23], [24]. These studies considered mean, minimum and maximum LST, not LST for day and night as in our analysis. However, in both studies minimum LST was excluded from multivariable analysis which is in parallel to the exclusion of our LST-night variable. Maximum LST was significantly negatively associated with infection which is in agreement with the significant LST-day variable in our final model. One study from Uganda found that *A. lumbricoides* prevalence is <5% where maximum LST exceeds 36–37°C [21] and a study conducted in 20 schools in the Chad predicted no *A. lumbricoides* prevalence in areas where mean LST exceeds 37 °C [25].

Denser vegetation, as indicated by a higher EVI, showed a strong positive association with *A. lumbricoides* infection in univariable analysis, which, however, turned non-significant when including other variables in the multivariable model. Our significant univariable result for EVI is in line with multivariable results from former studies [23], [24], [58]. The non-significance of EVI in multivariable analysis is likely due to differences in local conditions compared to the former studies where EVI showed a significant association.

The non-linear relationship of rainfall with infection can be explained based on the life cycle of *A. lumbricoides*. Rainfall is an important determinant of larval development because humidity, soil moisture, and land surface temperature have a strong impact on embryonation [22]. Laboratory studies showed that higher humidity facilitates larval development [59], [60]. A multiple regression analysis of rainfall, number of wet days and ambient temperature in Sri Lanka found a significant association between increased number of wet days per month and higher rates of *A. lumbricoides* infections [61]. These findings are in line with the first part of our predicted non-linear curve showing higher infection odds when rainfall increases. However, another laboratory study showed that the development of eggs located on an extremely wet soil surface was delayed due to evaporation and the resulting low temperatures [59]. Since, in contrast to hookworm larvae, *A. lumbricoides* eggs are non-motile [12], they are directly exposed to rainfall and LST on the soil surface. Crompton states that eggs may be washed away by rainfall, too [5]. Increased rainfall can lead to a leaching effect and eggs are washed to deeper regions of the soil [62]. All these findings indicate that up to a certain amount, increasing rainfall supports larval development but that too much rain can delay larval development and thus reduce transmission.

Regarding the examined possible confounding variables, our univariable and multivariable results indicate that low SES and bad sanitary conditions in and around the household are risk factors for *A. lumbricoides* infection which is in line with former publications [12], [14], [27], [28], [30], [32]. The non-linear association between age and *A. lumbricoides* infection describing a higher risk in children and an infection peak in later childhood, is in line with former epidemiological studies [3]–. Although none of these variables substantially confounded the associations of *A. lumbricoides* infection with environmental variables, it is important to consider such factors when planning interventions or further studies on a smaller scale, e.g. the community level.

In the literature, non-linear associations between environmental variables and STH infection have rarely been analyzed in a multivariable context. Brooker et al. [21] predict the prevalence of various STH infections with generalized additive models in a spatial analysis in Uganda and found that the predicted prevalence of *A. lumbricoides* infection showed non-linear relationships with LST and rainfall. A case study in Cameroon observed non-linear associations between environmental variables (LST, rainfall and vegetation) and *A. lumbricoides* infection in scatterplots of prevalence and environmental data [22]. Our results suggest that the MFP procedure can be effectively used as a multivariable parametric approach to detect non-linear associations between environmental data and *A. lumbricoides* infection. Especially when a turning point within a non-linear prediction is detected, such as for rainfall in our study, the MFP procedure can provide new insights into the relationship between environmental conditions and STH infection. A more precise understanding of such relationships could play an important role in the future prediction of high prevalence areas to be targeted for interventions.

However, non-linear analysis also has its limitations. Transformed variables are not directly interpretable, and thus hard to generalize and compare between studies. Moreover, fractional polynomials are very sensitive to outliers and over fitting, and transformations can often be due to extreme observations. To avoid this, it is very important to analyze transformed variables for outliers and define lower p-values for the function selection procedure when non-linear models are compared. Besides that, it is recommended to analyze non-linearity only if prior knowledge for non-linear relationships exists [54].

One problem when assessing STH infection by Kato-Katz (and most other microscopy based techniques) is the low sensitivity of the method. This is best compensated by the examination of more than one stool specimen, which was logistically impossible in our study. Instead, we examined two separate Kato-Katz slides from the same sample. Although this should have increased sensitivity, we have inevitably missed some of the lighter infections. However, the Kato-Katz examination of a single stool specimen shows a better sensitivity for the detection of *A. lumbricoides* infection than for other STH infections [63], [64].

Another limitation of our study is the lack of information concerning soil types in our study sites, which could be important in the context of rainfall and the survival of *Ascaris* eggs. Beaver states that *A. lumbricoides* infections were more common in regions with clayey soils [65], [66]. Sandy soils are more permeable and are unable to keep moisture. Moreover, in sandy soils eggs are more likely to be washed down to deeper soil strata. Furthermore we are unable to account for seasonal aspects of rainfall or the intensity of rainfall within a short time. Gunawardena et al. [61] found out that the number of wet-days per month were more significantly associated with *A. lumbricoides* infection than the total amount of rainfall. In this context they state that heavy rains facilitate the scattering of eggs both vertically and horizontally, whereas steady rainfall helps to maintain soil moisture.

Although our final model was able to account for most of the spatial autocorrelation in the raw *A. lumbricoides* infection data (Figure 5), the figure also shows some remaining positive spatial autocorrelation in the deviance residuals of our final model. This might have influenced the variance estimates of the model and in turn might have led to spuriously low p-values for some of our environmental variables. Therefore, these p-values should be interpreted with caution.

To conclude, *A. lumbricoides* infection was associated with several environmental, socio-demographic and sanitary factors in univariable analyses. Of these, mean annual rainfall, mean annual LST-day, age, SES and latrine coverage remained significant in multivariable analysis. MFP models can be used as an effective statistical tool to get a better understanding of the – often non-linear - relationship between environmental factors and *A. lumbricoides* infection. Future studies should therefore consider potential non-linear relationships between environmental factors and STH in a multivariable context to yield more precise predictions. However, if data are available, socio-demographic and sanitary conditions should also be considered, especially when planning interventions.

## Supporting Information

Checklist S1 **STROBE checklist.**
(DOC)
